# Serosurvey of arthropod-borne diseases among shelter dogs in the Cumberland Gap Region of the United States

**DOI:** 10.1186/s12917-020-02440-1

**Published:** 2020-06-30

**Authors:** Gilbert Patterson, Matthew Tanhauser, Paul Schmidt, Dawn Spangler, Charles Faulkner, Vina Faulkner, Daniel Kish, Karen Gruszynski, Hemant Naikare, Michele D. Coarsey, Ashutosh Verma

**Affiliations:** 1 College of Veterinary Medicine, Lincoln Memorial University, 6965 Cumberland Gap Parkway, Harrogate, TN USA; 2 Center for Human and Animal Health in Appalachia, College of Veterinary Medicine, Lincoln Memorial University, 6965 Cumberland Gap Parkway, Harrogate, TN USA; 3 Center for Infectious, Zoonotic and Vector-borne diseases, College of Veterinary Medicine, Lincoln Memorial University, 6965 Cumberland Gap Parkway, Harrogate, TN USA; 4 Tifton Veterinary Diagnostic and Investigational Lab, College of Veterinary Medicine, University of Georgia, 43 Brighton Road, Tifton, GA USA

**Keywords:** Shelter animals, Rocky Mountain spotted fever (RMSF), Anaplasmosis, Ehrlichiosis, Lyme disease, Canine heartworm, Cumberland, Gap region

## Abstract

**Background:**

The Cumberland Gap Region (CGR) of the United States is a natural corridor between the southeastern, northeastern, and midwestern regions of the country. CGR has also many species of ticks and mosquitos that serve as competent vectors for important animal and human pathogens. In this study, we tested dogs from six different animal shelters in the CGR for Rocky Mountain spotted fever (RMSF), anaplasmosis, Lyme disease, canine ehrlichiosis and canine heartworm disease.

**Results:**

Sera from 157 shelter dogs were tested for antibodies to RMSF agent, *Rickettsia rickettsii*, using an indirect immunofluorescence assay. Sixty-six dogs (42.0%) were positive for either IgM or IgG, or both IgM and IgG antibodies to *R. rickettsii*. Moreover, the same set of sera (*n* = 157) plus an and additional sera (*n* = 75) from resident dogs at the same shelters were tested using the SNAP 4Dx Plus. Of 232 dogs tested, two (0.9%) were positive for antibodies to *Anaplasma phagocytophilum/A. platys*, nine (3.9%) were positive for antibodies to *Borrelia burgdorferi*, 23 (9.9%) for positive for antibodies to *Ehrlichia canis/E. ewingii*, and 13 (5.6%) were positive for *Dirofilaria immitis* antigen. Co-infection with two or more etiologic agents was detected in five animals. Three dogs had antibodies to both *B. burgdorferi* and *E. canis/E. ewingii*, and two dogs were positive for *D. immitis* antigen and antibodies to *B. burgdorferi* and *E. canis/E. ewingii*.

**Conclusions:**

Shelter dogs in the CGR are exposed to a number of important vector-borne pathogens. Further studies are required to ascertain the roles these animals play in maintenance and transmission of these pathogens.

## Background

Focused surveillance efforts can provide vital clues about the movement and distribution of diseases between distinct regions of the United States. The Cumberland Gap Region (CGR) in the southeastern United States represents an ideal location for such a disease surveillance study as it is located at the intersection of the states of Tennessee, Kentucky, and Virginia. The ‘Cumberland Gap’ is a V-shaped passage across the Appalachian Mountains that has been used for millennia by populations of animals and humans migrating between the northeastern, midwest, and southeastern United States. Additionally, the CGR serves as an ecological transition zone between several distinctive geophysical regions of the United States, including the Appalachian Plateau, Blue Ridge, and Piedmont [6]. The region has a temperate climate, ample rainfall, a diverse wildlife population and numerous species of mosquitos and ticks. Moreover, several species of ticks and mosquitos found in the CGR are competent vectors for a number of important disease of dogs and humans.

The tick-borne diseases that are frequently tested in US dogs are Lyme disease (caused by *Borrelia burgdorferi*), ehrlichiosis (caused by *Ehrlichia canis* or *E. ewingii*), and anaplasmosis (caused by *Anaplasma phagocytophilum* or *A. platys*) [2]. Lyme disease in dogs is characterized by fever, lethargy, anorexia, swollen joints, lameness, lymphadenopathy, and in a sub-set of cases, glomerulonephritis [11]. Canine ehrlichiosis and anaplasmosis are similarly characterized by protean manifestations, such as fever, anorexia, myalgia, and thrombocytopenia. In severe cases of ehrlichiosis and anaplasmosis, hemorrhages (such as epistaxis) and death may result [15].

Rocky Mountain spotted fever (RMSF) is also a tick-borne disease, but is less frequently tested in dogs; however, its increasing frequency of detection in recent decades supports recognition as an emerging zoonotic disease [17]. Caused by *Rickettsia rickettsii*, RMSF in dogs can cause severe vasculitis and a range of clinical presentations including ocular lesions, neurologic dysfunction and arthropathy.

Another arthropod-borne disease that is frequently tested in dogs is canine heartworm disease. Caused by *Dirofilaria immitis,* canine heartworm is a mosquito borne disease facilitated by an obligate relationship between microfilaremic dogs hosting the parasite and vector competent mosquito species required for its development to infectivity and subsequent transmission to susceptible canine hosts. Canine heartworm is a significant cause of morbidity and heart disease in dogs world-wide, and a recent American Heartworm Society sponsored survey indicated that the incidence of canine heartworm disease has trended upwards and the number of infected dogs per clinic rose 21% between 2013 and 2016 [1].

Dogs housed in animal shelters are well recognized as a population at increased risk of carrying or spreading a variety of important diseases to both animals and humans. Given their origin as unwanted animals and due to unsanitary living conditions in shelters, high population density, stress, and exposure to rodents and arthropod vectors, shelter dogs are especially useful sentinels for many zoonotic and vector-borne diseases [16].

The purpose of this study was to conduct a serological survey of arthropod-borne diseases in shelter dogs from CGR, an area that represents a key transport corridor between distinct regions of the United States. To that end, canine sera from six different animal shelters located in the CGR were tested for RMSF, anaplasmosis, Lyme disease, canine ehrlichiosis and canine heartworm disease.

## Results

### RMSF testing

Serum samples (*n* = 157) were tested for antibodies to *Rickettsia rickettsii*, the causative agent of Rocky Mountain spotted fever. An immunofluorescence assay (IFA) was used to detect anti-*Rickettsia rickettsii* immunoglobulin (Ig)-M and IgG antibodies. Out of 157 tested sera, 23 sera were positive for pathogen-specific IgM, 27 were positive for pathogen-specific IgG and 16 sera were positive for both IgM and IgG. Overall, 66 dogs (42.0%, 95 CI: 34.2–50.2%) were positive for either IgM, IgG, or IgM and IgG antibodies. RMSF seropositivity ranged between 17.4–100% among the six shelters (Table 1).

**Table 1:** Results of indirect immunofluorescence assay for *Rickettsia rickettsii*

Shelter ID	Number of dog sera tested	Number of IgM^a^ positive	Number of IgG^b^ positive	Number of both IgM^a^ and IgG^b^ positive	Total number of positives and percent positive	95% Confidence interval (CI)
Shelter KR	80	10	16	8	34 (42.5%)	31.5–54.1%
Shelter KW	23	2	9	4	15 (65.2%)	42.7–83.6%
Shelter CC	15	3	0	2	5 (33.3%)	11.8–61.6%
Shelter PW	23	3	1	0	4 (17.4%)	5–38.8%
Shelter HL	1	1	0	0	1 (100%)	2.5–100%
Shelter BC	15	4	1	2	7 (46.7%)	21.3–73.4%
TOTAL	**157**	**23**	**27**	**16**	**66 (42%)**	**34.2–50.2%**

*IgM* Immunoglobulin M, *IgG* Immunoglobulin G

^*a*^ an IgM titer of ≥16 denotes a positive result

^*b*^ an IgG titer of ≥64 denotes a positive result

Based on the guidelines for the interpretation of RMSF antibody titers, 23 dogs had IgM titer (≥16) without presence of IgG suggesting infection within the past 2–4 weeks, four dogs had IgM titers ≤8 and IgG titers ≥512, indicating infection within 2 weeks to 3 months, 13 dogs had elevated titers for both antibodies, indicating past infection within 4–8 weeks, and 23 dogs had IgM titers ≤8 and IgG titers = 64–256, indicating past infection. Only three out of 66 seropositive dogs had high IgM titer along with a very high IgG titer (≥512), suggesting recent or active infection.

### Arthropod-borne pathogens testing

A highly sensitive and specific ELISA-based rapid assay (SNAP 4DX Plus), was used to screen canine sera for antigen or antibodies to six arthropod-borne pathogens. This assay detects *Dirofilaria immitis* antigen, and antibodies to *Anaplasma phagocytophilum*/*A. platys*, *Borrelia burgdorferi*, *Ehrlichia canis*/*E. ewingii*.

**Fig. 1:**
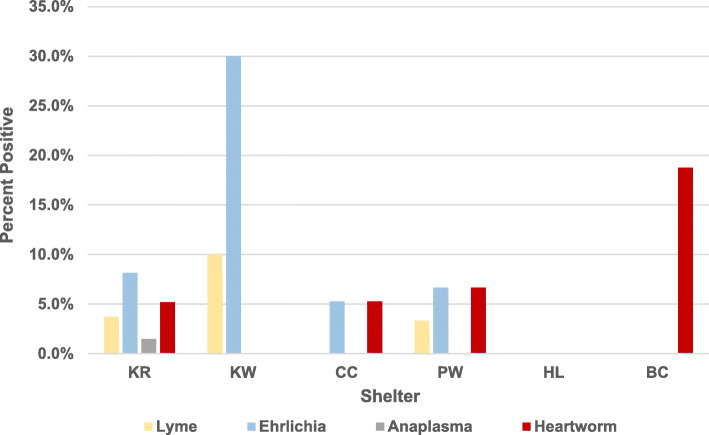
Shelter-wise distribution of seropositive animals. Graph showing percent of seropositive dogs for Lyme Borreliosis, Ehrlichiosis, Anaplasmosis and Heartworm in six shelters (KR, KW, CC, PW, HL, BC) studied in this work. Number of dog sera tested from each shelter is as follows: KR = 135, KW = 30, CC = 19, PW = 30, HL = 2, BC = 16

The same set of canine sera (*n* = 157) and additional sera from animals housed in the same shelters (*n* = 75) were tested using the SNAP 4Dx Plus. Of 232 dogs tested, two (0.9, 95% CI: 0 -2.1%) were positive for anaplasmosis, nine (3.9, 95% CI: 1.4–6.4%) for Lyme disease, 23 (9.9, 95% CI: 6.1–13.7%) for ehrlichiosis, and 13 (5.6, 95% CI: 2.6–8.6%) for heartworm disease (Table 2). SNAP 4Dx Plus test results varied by shelter and disease ranging from 0 to 30% (Fig. 1).

**Table 2:** SNAP 4DX Plus test results (*n* = 232)

Pathogen(s) tested	Number positive	Percent positive	95% Confidence interval (CI)
*Anaplasma phagocytophilum* and/or *A. platys*^*a*^	2	0.9%	0–2.1%
*Borrelia burgdorferi*	9	3.9%	1.4–6.4%
*Ehrlichia canis and/or E. ewingii*^*a*^	23	9.9%	6.1–13.7%
*Dirofilaria immitis*^*b*^	13	5.6%	2.6–8.6%

^a^ detection of pathogen-specific antibodies

^b^ detection of pathogen-specific antigen

Five dogs tested positive for more than one pathogen. Three dogs were seropositive for both *Borrelia burgdorferi* and *Ehrlichia canis*/*E. ewingii*, while two dogs tested positive for *Borrelia burgdorferi, Ehrlichia canis/E. ewingii* and *D. immitis*.

Of note, no dogs < 1-year old (*n* = 20) tested positive using the SNAP 4Dx Plus test, whereas 10 dogs < 1-year old (*n* = 17) tested positive for antibodies to RMSF. The distribution of male and female positive dogs was very similar for the five vector-borne diseases. Despite variation in age and shelter results, analyses did not show any statistically significant differences between the groups.

## Discussion

Ticks transmit multiple and diverse pathogens that can cause a range of zoonotic diseases, including Rocky Mountain spotted fever (RMSF), Lyme disease, ehrlichiosis, and anaplasmosis. Vertebrate animals, including dogs, play an integral role in the life cycle of tick species, and also live and interact closely with humans. RMSF is a common and potentially devastating rickettsial disease in both humans and dogs. Although RMSF is reported throughout the United States, it is most commonly reported from North Carolina, Tennessee, Missouri, Arkansas, and Oklahoma [3]. In the present study, we found an overall seropositivity of 42% for RMSF, indicating some history of exposure to or active infection with *Rickettsia rickettsii.* This level of seropositivity is substantially higher than those found in comparable recently published studies of dog population in the United States that were undertaken in Arizona (5.1%), and Rhode Island (14.4%) [9, 19]. This information provides ample cause for concern among veterinarians and other public health professionals in the CGR, considering the important role dogs play in the emergence and spread of *R. rickettsii.*

Lyme disease historically has been mostly restricted in the northeastern and upper midwestern United States, but CGR seems to be at the leading edge of Lyme disease expansion. In this study, 3.84% of dogs were seropositive for antibodies to the Lyme disease spirochete, *Borrelia burgdorferi*. In a previous study, 3.3% of dogs from southern United States were seropositive for *B. burgdorferi* [10, 14]. This information is important as earlier studies indicate that seroreactive dogs are effective sentinels for human Lyme disease risk [5, 13]. The seroprevalence of *Ehrlichia* spp. in our study was 9.82%, which is higher than the previously reported 3.2% in dogs from southeastern US [12] or 5.2% (*E. ewingii*) and 2.3% (*E. canis*) from southern US [14]. The seroprevalence of *Anaplasma* spp. in our study was 0.85%, which is lower than the previously reported prevalence of 2% (*A. platys*) and 2.1% (*A. phagocytophilum*) in southern US [14] and similar (0.9%) in southeastern US [12].

The 5.56% seroprevalence of canine heartworm infection detected in our sample is comparable to that previously documented in a study of sheltered dogs in the CGR [18]. In that study of 655 dogs from regional animal shelters, 35 (5.3%) were positive for canine heartworm antigen. Taken together, our study and Watlington’s [18] previous study indicate substantially higher prevalence of infection in sheltered dogs compared to the 1.8% prevalence reported by the Companion Animal Parasite Council for pet dogs in the CGR counties for 2017 [4]. This is not unexpected as the occurrence of heartworm in canine shelter populations likely reflects animals that have not benefited from regular veterinary preventive care or consistent use of effective prophylactic medication for protection against infective larvae transmitted by mosquito vectors. Heartworm positive, microfilaremic dogs from shelter populations are an important reservoir for infection of vector competent mosquitos and subsequent transmission to unprotected pet dogs.

Both 2-way and 3-way co-infections were observed in our study. In three sampled dogs, antibodies to both *B. burgdorferi* and *E. canis/ewingii*, were observed, while two dogs were seropositive for *B. burgdorferi*, *E. canis/ewingii* and *D. immitis*. Co-infections often complicate clinical diagnosis and can occur from simultaneous or sequential exposure to different tick species or in cases where more than one pathogen is transmitted by a single tick species [7].

While this study represents sampling done only in shelter dogs, it raises concerns for the prevalence of these arthropod-borne diseases in owned pets as well. Generally, owned dogs have an increased chance of receiving veterinary care such as flea/tick/heartworm preventatives that may reduce the transmission of arthropod-borne diseases. However, all dogs in this socioeconomically backward region are likely to have the same risk in the absence of routine tick control and heartworm prevention. Additionally, many pet owners in this region cannot afford to get their pets altered, leading to more roaming behavior in pets and increased risk of exposure and/or transmission.

There are some limitations in our study: although SNAP 4DX Plus is a rapid and easy-to-perform test, the analytes utilized in the test for detecting antibodies for *Ehrlichia canis/E. ewingii* and *Anaplasma phagocytophilum/A. platys* do not differentiate between the individual species of *Ehrlichia* and *Anaplasma* and thus limit conclusions on the individual species involved in infection. In addition, the test does not quantify the antibodies, which is especially important for deciding the treatment plan for Lyme disease seropositive dogs in endemic areas. Another limitation of this study is that travel histories of these dogs were not obtained, so it is not possible to say that exposure to infected ticks was restricted to the location of the shelter of origin.

## Conclusions

In summary, our results show that all arthropod-borne diseases examined in this study are present in the Cumberland Gap Region and could potentially pose risk to dogs and humans throughout the area. Additionally, knowledge of vector distributions may be useful in predicting the pattern of disease associated with particular vector species and may aid diagnostic, prevention, and control efforts. Together, these data will provide veterinarians with a heightened awareness of the vector-borne disease agents common in their practice areas, and elevate their consideration so that appropriate diagnostic testing and antibiotic therapy may be performed, if indicated.

## Methods

### Ethics statement

This study was conducted on blood samples previously collected for medical surveillance under a protocol that was approved by the Institutional Animal Care and Use Committee at the Lincoln Memorial University. All sample collections were handled in strict accordance with U.S. Animal Welfare Act, its amendments and associated Regulations (https://www.nal.usda.gov/awic/animal-welfare-act). Memoranda of Understanding (MOUs) established between all parties provided informed consent for collection of blood samples and analysis of clinical disease.

### Sample collection

**Fig. 2:**
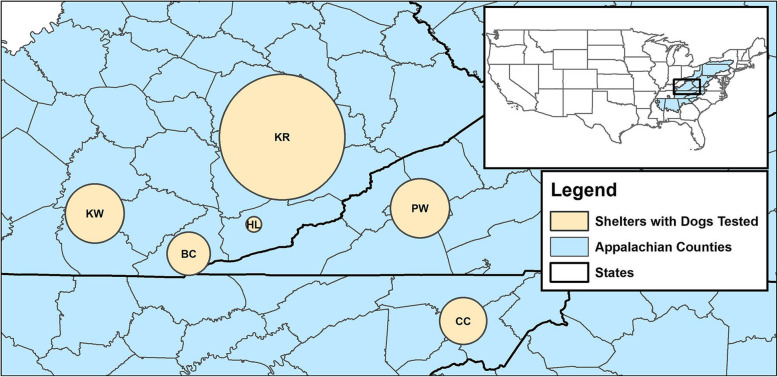
Location of animal shelters. Map showing the location of six animal shelters in Kentucky, Virginia and Tennessee states of the Southeastern United States of America from where 232 dogs included in this study were housed. Map created using ArcMap 10.6 (Esri, Redlands, CA). Sizes of circles are proportional to the number of dogs tested from each shelter

Dogs from six shelters located in three states (Tennessee, Kentucky and Virginia) were sampled in this study from April 2017 to Mar 2018 (Fig. 2). All animals included in this study were clinically healthy. Blood samples were collected shortly after their arrival at the Lincoln Memorial University - College of Veterinary Medicine’s Small Animal Medical Center under the Shelter Outreach for the Appalachian Region (SOAR) program. The SOAR program provides spay/neuter services and medical care to unowned animals. These animals were clinically healthy and the number of animals included in the study from each shelter depended solely on the number of animals that were brought to the LMU-CVM Small Animal Medical Center for basic veterinary care. Blood samples (1.5 mL) were drawn by venipuncture and collected in IDEXX Vacuette SST-Serum Separator Tube. Tubes were then centrifuged and serum pipetted off, aliquoted and stored at -20 °C. Demographic data including sex, age, breed, and shelter were recorded in Excel (Microsoft, Redmond, WA). The dogs were released back to the shelter to be moved into their adoption program. No animals were euthanized on this protocol.

### RMSF testing

The serum samples were tested by immunofluorescence assay (IFA) to detect anti-*Rickettsia rickettsii* immunoglobulin (Ig)-M and IgG antibodies using *Rickettsia rickettsii* as antigen. The test was performed by the Infectious Diseases Laboratory, University of Georgia (Athens, Georgia, USA) as per their proprietary procedures. The interpretation of RMSF antibody titers is as follows: IgM titers ≤8 and IgG titers < 64 are seronegative for antibody to RMSF. An IgM titer ≥16 is considered seropositive and compatible with a recent RMSF infection. An IgG titer ≥64 is considered seropositive. An elevated IgM titer (≥ 16) without presence of IgG suggests infection within the past 2–4 weeks. A very high IgG titer (≥ 512) along with the high IgM titer suggests recent or active infection. Elevated titers for both antibodies suggest infection occurred within the past 4–8 weeks. IgM titers ≤8 and IgG titers = 64–256 suggest past infection. Some IgG titers at this level may persist for years. This also may represent an early infection. A four-fold or greater increase in the IgG titer on a recheck sample in 2 weeks indicates an active infection. IgM titers ≤8 and IgG titers ≥512 are compatible with a recent RMSF infection of between 2 weeks to 3 months’ duration. Because IgG titers ≥512 rarely persist longer than 1 year, such seropositive dogs have been most likely infected in a recent season when the sample was collected [8].

### SNAP 4DX plus

The SNAP 4DX Plus (IDEXX Laboratories Inc., Westbrook, Maine, USA), an ELISA-based rapid assay, was performed to test the presence of *Dirofilaria immitis* antigen, and antibodies to *Anaplasma phagocytophilum*/*A. platys*, *Borrelia burgdorferi*, *Ehrlichia canis*/*Ehrlichia ewingii*, following the manufacturer’s recommendations. Briefly, three drops of canine serum were mixed with two drops of the conjugate in a fresh tube, and added to the sample well of the SNAP device. As soon as the sample-conjugate mixture reaches the activation circle, the device is activated. Results were manually read 8 min after activating the device. Color development in sample spots indicates a positive reaction. The sensitivity and specificity of the SNAP 4DX Plus test in dogs is 99 and 99.3% for heartworm disease, 90.3 and 94.3% for anaplasmosis, 94.1 and 96.2% for Lyme disease, and 97.1 and 95.3% for ehrlichiosis (IDEXX Laboratories, Inc.).

### Statistics

Variables and test results were recorded in Excel (Microsoft, Redmond, WA). Briefly, Chi-Square Test or Fisher’s Exact Test were performed for the variables: sex (M, F), age (< 1-year-old, ≥ 1 year old), and the six shelters for the different tests. Analysis was performed using SPSS 25 (IBM, New York).

## Data Availability

The datasets used and/or analysed during the current study are available from the corresponding author on reasonable request.

## Abbreviations

CGR: Cumberland Gap Region
RMSF: Rocky Mountain spotted fever
IgG: Immunoglobulin G
IgM: Immunoglobulin M
US: United States
AHS: American Heartworm Society
IFA: Immunofluorescence Assay
CI: Confidence Interval
CDC: Centers for Disease Control and Prevention
CAPC: Companion Animal Parasite Council
MOUs: Memoranda of Understanding
SOAR: Shelter Outreach for the Appalachian Region
LMU-CVM: Lincoln Memorial University College of Veterinary Medicine
